# Thulium laser vaporesection of prostates with volume exceeding 100 cm^3^ as an alternative to HoLEP and ThuLEP

**DOI:** 10.1093/jscr/rjac441

**Published:** 2023-05-29

**Authors:** Nahuel Paesano, Gonzalo Castañeda, Alicia Maccagno, Paulo Caldas, Gilberto Chechile

**Affiliations:** Instituto Médico Tecnológico, Barcelona, Spain; Prostate Institute Barcelona, Barcelona, Spain; Department of Urology, Cima Sanitas Hospital, Barcelona, Spain; Instituto Médico Tecnológico, Barcelona, Spain; Prostate Institute Barcelona, Barcelona, Spain; Department of Urology, Cima Sanitas Hospital, Barcelona, Spain; Statistics Area, National University of Cordoba, Córdoba, Argentina; Department of Urology, Hospital Regional do Oeste, Chapecó, Brazil; Instituto Médico Tecnológico, Barcelona, Spain; Prostate Institute Barcelona, Barcelona, Spain; Department of Urology, Cima Sanitas Hospital, Barcelona, Spain

**Keywords:** BPH, prostate, benign prostatic hyperplasia, treatment, thulium laser, vaporesection

## Abstract

The aim of this study is to evaluate the outcomes of thulium laser vaporesection of prostates with volume exceeding 100 cm^3^. In the present prospective study, patients with infra-vesical urinary obstruction due to a prostate with volume exceeding 100 cm^3^ underwent endoscopic vaporesection using thulium laser. In this procedure, prostate chips were resected without morcellation. The technical aspects of surgery, admission time, post-operative catheter time and post-operative complications were analyzed. Flowmetry was performed combined with prostatic ultrasound in the follow-up. Between March 2010 and November 2018, 156 cases with benign prostatic hyperplasia (BPH; volume >100 cm^3^) were treated. The mean patient age was 67.8 years (48.4–86.6 years), and the mean prostatic volume was 137 cm^3^ (100–436 cm^3^). The mean length of hospitalization was 1.48 days (1–8 days), and the mean post-operative catheter time was 5.1 (1–17). Three cases (1.9%) required readmission due to hematuria. The mean follow-up time was 31.2 months (standard deviation = 27.7). Urethral stricture was observed in 14 cases (9%), with bulbar urethra being the most frequent finding. Urinary tract infection was observed in 11 cases (7.1%), and urinary incontinence was observed in 5 cases. The mean peak urinary flow at 12 and 24 months was 26.9 ± 12.5 and 23.9 ± 11.7 ml/s, respectively, and the mean urinary flow during the final follow-up at 41 months was 21.6 ml/s. Thulium laser vaporesection is a valid alternative to open prostatectomy, HoLEP and ThuLEP in patients with large BPH. Urinary flow remained elevated throughout the follow-up.

## INTRODUCTION

Benign prostatic hyperplasia (BPH) is one of the most frequent medical conditions in men over 60 years of age, and its prevalence increases with the progression of age [1, 2]. Since decades, transurethral resection (TUR) and open prostatectomy (OP) have been the treatments of choice for BPH-associated with drug refractory lower urinary tract symptoms (LUTS). However, the selection of approaches depends on prostate volume [3]. Recent advances in medical technologies and introduction of lasers have decreased morbidity, shortened post-operative catheterization time and reduced hospital stay while achieving functional outcomes comparable to the conventional modalities [4, 5].

Laser surgical techniques for BPH management include vaporization, vaporesection and enucleation, depending on the size of the prostate and the type of laser used [6]. In recent years, for prostates with volume exceeding 100 ml, holmium:YAG or thulium:YAG laser enucleation has been used as an alternative to OP, with excellent functional outcomes and reduced incidence of complications [7, 8].

For surgeons with the experience of TUR, the learning curve of vaporesection is shorter than that of enucleation, and the high coagulation capacity allows the surgeon to operate on much larger prostates with minimal risk of bleeding [9, 10].

According to the clinical guidelines of the European Association of Urology, thulium laser vaporesection offers results equivalent to TUR, with advantages of shorter post-operative catheterization time, shorter hospital stays, and lower incidence of complications (level of evidence 1b) [6].

In the present study, we analyzed the outcomes on our series of patients with prostate volumes >100 cm^3^ with LUTS by means of the vaporesection technique with a 200-W thulium:YAG laser.

## MATERIALS AND METHODS

Between March 2010 and November 2018, 156 patients having prostates with volume exceeding 100 cm^3^ were treated with thulium laser vaporesection. The mean patient age was 67.8 years (48.4–86.6 years).

The Quanta System® Cyber™ 200 W Thulium: YAG system (Quanta System. Varese, Italy) operating at a wavelength of 2010 μm was used. A 1000-μ fiber with continuous emission and power between 150 and 200 W was employed.

### Inclusion criteria

Inclusion criteria were prostate >100 cc in patients with drug refractory LUTS or complications derived from BPH (e.g. recurrent urinary tract infections and use of urethral catheter).

### Exclusion criteria

The exclusion criteria were pelvic radiotherapy and neurogenic bladder dysfunction.

All cases in this series were performed by the same surgeon. The bladder was accessed using a 26F double-sheath Iglesias-type resectoscope under continuous irrigation with physiological saline. The bladder was explored to identify the location of the ureteral meatus and to detect any previously undiagnosed bladder pathologies, such as tumor or lithiasis. Subsequently, the prostatic urethra and the presence of lateral or middle lobe were recognized.

The distal limit of treatment was the verumontanum, which was preserved. The cutting power of the 1000-μ front fiber was set between 150 and 200 W, depending on the size of the prostate. Coagulation was predetermined at 50 W.

When the middle lobe was present, surgery was initiated with two incisions at Hours 5 and 7 until reaching the capsule. The direction was always from the bladder neck toward the pre-verumontanum. The initial resection of the middle lobe allowed a greater amplitude for the subsequent resection of the lateral lobes.

The resection of the lateral lobes was initiated by making an incision at Hour 1 on the left lobe and another at Hour 11 on the right lobe.

The depth limit was established by the presence of the prostatic capsule. The fiber moved vertically and horizontally. For resection of the apical lobes, the power was reduced to 120 W so as not to damage the pre-sphincteric area and the sphincter.

At the end of surgery, the verumontanum had to be undamaged. Fragments were removed from the bladder with a Toomey syringe and hemostasis was checked.

Finally, a 20 Fr. bladder catheter was inserted with continuous saline flushing.

The patients were discharge within 24 hours if there were no complications. The catheter was removed before discharge, or at 48–72 hours on an outpatient basis.

Follow-ups were performed with flowmetry at 3 and 6 months and then annually. Prostatic ultrasound was performed at each annual follow-up.

### Statistical methodology

With the data collected, central tendency and dispersion measures were calculated for continuous variables (mean and standard deviation (SD)) as well as absolute and percentage distributions for categorical variables. To compare means, a student’s *t*-test was applied (after the Shapiro–Wilk normality test) and, in the case of correlations between categorical variables, the chi-squared test was applied, using a confidence level of 0.05. Kaplan-Meier curves were performed to evaluate follow-up data.

## RESULTS

At the time of surgery, 48 patients (31%) had bladder catheterization. The mean catheterization time prior to surgery was 45.3 days (SD = 35.1; range: 2–120 days).

The most used pre-operative medical treatment was alpha-blockers alone, or in conjunction with 5-alpha reductase inhibitors. In eight cases (5.1%), there was a history of previous prostate surgery with TUR (one case), OP (two cases) or some type of laser (five cases).

The mean PSA was 5.97 ng/ml (SD: 3.87; range: 0.7–24.8). Six cases (3.8%) were receiving treatment with decoagulants, which were discontinued 4–7 days before surgery. Twelve patients (7.7%) had a bladder lithiasis that was treated simultaneously with the same energy laser.

The mean prostatic volume was 134 cm^3^ (SD: 44.2 cm^3^) with a range of 100–436 cm^3^. The mean maximum urinary flow before surgery was 8.78 ml/s (SD: 5.3; range: 2.3–17.5).

The mean surgery time was 110.7 minutes (SD: 38.9; range: 30–240). Means of 170.4 W (SD: 23.9; range: 150–200 W) and 584 507 J (range: 50 000–1000 000 J) were used.

Admission time was <24 hours in 104 cases (66.7%), 48 hours in 36 cases (23.1%) and >48 hours in 16 cases (10.2%) (of which, in 13 cases, the cause of prolongation of hospital stay was bleeding; in 1 case, it was fever that required observation; and in 2 cases, the prolongation of stay did not have to do with urological causes).

One patient (0.6%) with coagulation disorder had to undergo transfusion of two red blood cell concentrates in the immediate post-operative period.

In 39 cases (25%), the catheter was removed at discharge before 24 hours post-surgery. The mean catheterization time in cases discharged with catheter was 5.1 days (SD: 2.2; range: 1–14 days).

Three patients (1.9%) were readmitted in the first post-operative month due to hematuria. Bladder lavage was required in two cases, and in one case, endoscopic coagulation of blood areas was required.

Urinary tract infection was observed in 11 cases (7.1%) of which 1 required hospitalization.

The mean follow-up of the series was 41.3 months (SD: 32.5). In 82 operated patients (52%), follow-up was >2 years.

In the immediate post-operative period, some type of urinary incontinence was observed in 48 cases (30.7%). In 46 patients (29.5%), anticholinergics were used for a period of 1–3 months due to urinary urgency and urine leakage. In 20 cases (12.8%), pelvic floor physiotherapy (PFP) was used to resolve urinary incontinence.

In five patients (3.2%), incontinence was definitive. Of these, only one case required surgical correction with adjustable sub-urethral tape (Reemex®). In the remaining cases, urine leakage ranged between 20 and 40 g of urine per day, and they refused corrective surgery.

Urethral stricture was observed in 14 cases (9%). The location of the stricture was the bulbar urethra (10 cases), navicular fossa (3 cases) and bladder neck (1 case). The treatment used was urethral dilatation (seven cases), internal urethrotomy (five cases) and TUR of the bladder neck (one case).

**Figure 1:**
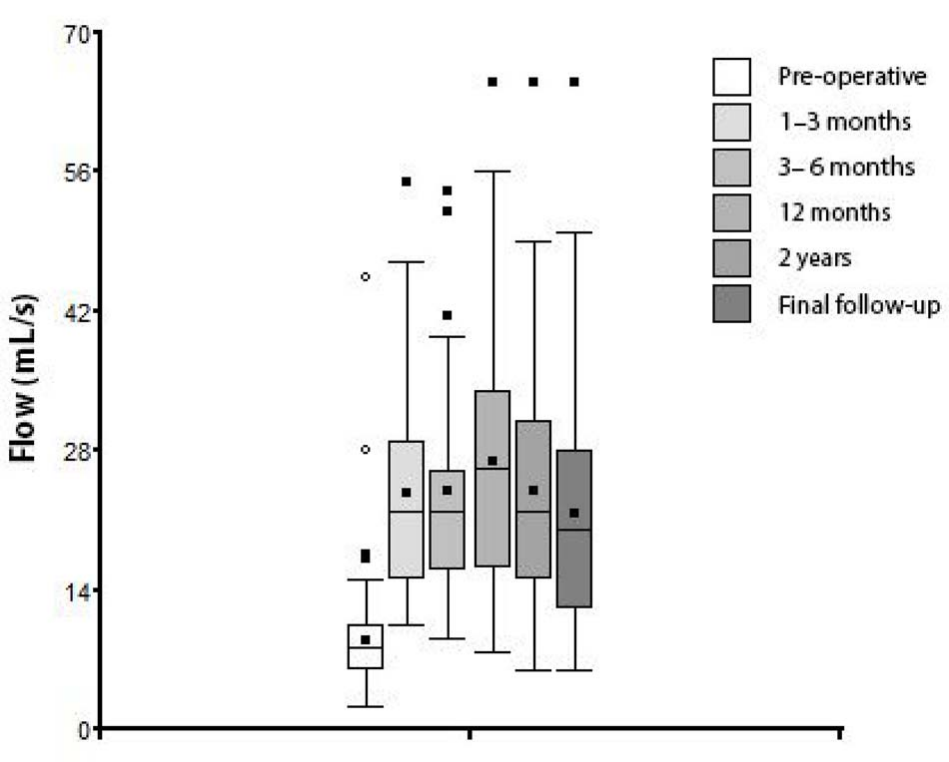
Box plot of peak urinary flow according to the time of measurement.

The evolution of peak urinary flow according to the time of measurement is presented in Table 1 and Fig. 1.

**Table 1:** Evolution of peak urinary flow according to the time of measurement

Time of measurement	Number of cases	Mean (ml/s)	SD	Minimum	Maximum
Pre-operative	110	8.78	5.15	2.30	17.5
Post-operative 1–3 months	44	23.7	10.3	10.5	55.0
Post-operative 3–6 months	45	23.9	10.7	9.20	54.0
Post-operative 12 months	72	26.9	12.5	7.70	65.0
Post-operative 24 months	67	23.9	11.7	6.00	65.0
Final follow-up at 41 months	104	21.6	11.4	6.00	65.0

The incidence of initial or definitive urine incontinence was not correlated with the pre-operative urinary flow (*P* = 0.37) or prostate volume (*P* = 0.35).

Likewise, the incidence of initial or definitive incontinence was not correlated with the technical aspects of surgery (watts (*P* = 0.93), joules (*P* = 0.19), previous PSA (*P* = 0.19), pre-operative catheter retention time (*P* = 0.60) and post-operative catheter retention time (*P* = 0.11)). However, the longer the duration of surgery, the higher the incidence of initial incontinence, and this correlation was statistically significant (*P* = 0.04).

The incidence of definitive incontinence was significantly reduced in patients who received PFP (*P* = 0.0007).

**Figure 2:**
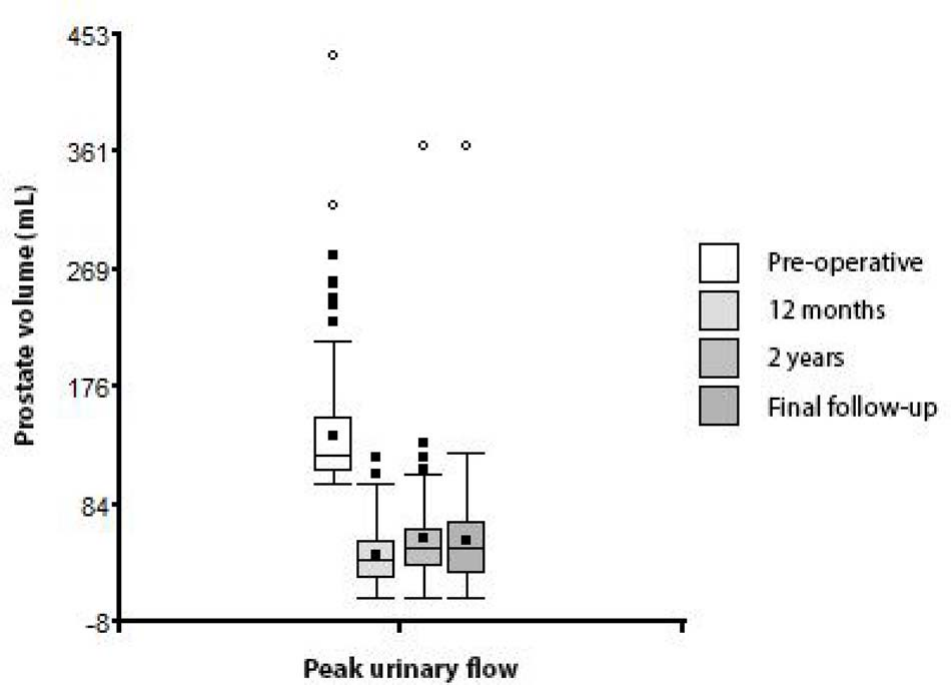
Box plot of prostate volume according to time of measurement.

Finally, the evolution of the prostate volume during the follow-up is represented in the Table 2 and Fig. 2.

**Table 2:** Evolution of prostate volume according to the time of measurement

Time of measurement	Number of cases	Mean (ml)	S.D.	Minimum	Maximum
Pre-operative	156	138	45.9	100	436
Post-operative 12 months	69	44.0	23.2	10	120
Post-operative 24 months	64	57.9	47.1	10	365
Final follow-up at 41 months	84	56.5	44.1	10	265

## DISCUSSION

For >50 years, TUR has been the most used technique for the treatment of LUTS caused by prostates with volumes <80 ml. In patients with larger glands, open surgery was the preferred option.

With the introduction of laser technology within the last 20 years, surgical techniques have undergone some modifications [5, 11]. The thulium laser was introduced in 2005 for the treatment of BPH, initially for vaporization and later for vaporesection and enucleation.

The vaporesection technique vaporizes and resects the prostate in a very similar way to TUR, so the learning curve is expected to be short [12, 13].

Thulium laser surgery has advantages, such as lower incidence of bleeding, shorter hospitalization time and less frequent post-operative bladder catheterization [10].

Regarding functional results, neither significant differences were observed in post-operative peak flow between conventional TUR and vaporesection nor differences were demonstrated in the incidence of complications [14].

In a meta-analysis study, in which 16 studies were evaluated, 9 of which were randomized, the results between thulium laser vaporesection and bipolar TUR were compared. In patients treated with vaporesection, the decreases in hemoglobin, hospital stay and catheter time were lower than in cases treated with bipolar TUR [15]. The incidence of complications in our series was similar to those observed by Rassweiler *et al*. with TUR [16].

The appearance of urinary incontinence in the post-operative period has a negative influence on the quality of life of the patients. Incontinence was observed in 3–9% of the cases operated with open surgery, in 2% of those treated with TUR and in 5–12% of those operated with holmium laser enucleation. Most of these patients recovered within the first year after the procedure [17–19].

In our series, the high incidence of transient urinary incontinence in the immediate post-operative period may be related to the high energy levels used (150–200 W), the extent of the resection (in the case of prostates >100 cm^3^) and transient thermal affectation of the external sphincter when acting in the area distal to the apical lobes. With the use of PFP and/or anticholinergics, incontinence was resolved in most cases.

In general, the TUR surgical option is limited to cases with prostate volumes <80 cm^3^, so cases with larger prostates should be treated with open surgery or with holmium and thulium laser enucleation. The latter technique has a longer learning curve, so thulium laser vaporesection allows us to benefit from the advantages of laser technology using a technique widely mastered by urologists, such as TUR.

In the series of Yu *et al*., with a mean prostatic weight of 60 cm^3^, the mean operative time was 44 minutes [20]. In our series, with a mean prostatic weight of 134 cm^3^, the operative time was 110.7 minutes.

In our series, 67% of the cases were discharged before 24 hours after surgery; and in 23%, the hospital stay was 48 hours. In the other series, admission time was >5 days [20].

Regarding the functional results, we observed that with the vaporesection technique, the improvement in peak flows was maintained during follow-up at 12, 24 and 41 months, with flows of 27, 24 and 22 ml/s, respectively.

These results are comparable to those observed by Zhang *et al*., who compared holmium and thulium laser enucleation; and by Becker *et al*., who performed thulium laser enucleation [21, 22].

## CONCLUSION

Thulium laser vaporesection is a valid option for the treatment of patients with BPH and enlarged prostates.

This modality can be considered a reliable alternative to the conventional prostate TUR as well as OP and offers several advantages such as shortened admission time and reduced bladder catheterization duration. Finally, in our experience, thulium laser vaporesection markedly improved peak urinary flow, which remained high throughout the follow-up period.
